# Deciphering lineage-relevant gene regulatory networks during endoderm formation by InPheRNo-ChIP

**DOI:** 10.1093/bib/bbae592

**Published:** 2024-11-13

**Authors:** Chen Su, William A Pastor, Amin Emad

**Affiliations:** Department of Electrical and Computer Engineering, McGill University, 845 Sherbrooke Street West, Montreal, Quebec H3A 0G4, Canada; Department of Biochemistry, McGill University, 845 Sherbrooke Street West, Montreal, Quebec H3A 0G4, Canada; The Rosalind and Morris Goodman Cancer Institute, 1160 Pine Avenue, Montreal, Quebec H3A 1A3, Canada; Department of Electrical and Computer Engineering, McGill University, 845 Sherbrooke Street West, Montreal, Quebec H3A 0G4, Canada; The Rosalind and Morris Goodman Cancer Institute, 1160 Pine Avenue, Montreal, Quebec H3A 1A3, Canada; Mila, Quebec AI Institute, 6666 St-Urbain Street #200, Montreal, Quebec H2S 3H1, Canada

**Keywords:** phenotype-relevant gene regulatory network, endoderm formation, RNA-seq, ChIP-seq

## Abstract

Deciphering the underlying gene regulatory networks (GRNs) that govern early human embryogenesis is critical for understanding developmental mechanisms yet remains challenging due to limited sample availability and the inherent complexity of the biological processes involved. To address this, we developed InPheRNo-ChIP, a computational framework that integrates multimodal data, including RNA-seq, transcription factor (TF)–specific ChIP-seq, and phenotypic labels, to reconstruct phenotype-relevant GRNs associated with endoderm development. The core of this method is a probabilistic graphical model that models the simultaneous effect of TFs on their putative target genes to influence a particular phenotypic outcome. Unlike the majority of existing GRN inference methods that are agnostic to the phenotypic outcomes, InPheRNo-ChIP directly incorporates phenotypic information during GRN inference, enabling the distinction between lineage-specific and general regulatory interactions. We integrated data from three experimental studies and applied InPheRNo-ChIP to infer the GRN governing the differentiation of human embryonic stem cells into definitive endoderm. Benchmarking against a scRNA-seq CRISPRi study demonstrated InPheRNo-ChIP’s ability to identify regulatory interactions involving endoderm markers *FOXA2*, *SMAD2*, and *SOX17*, outperforming other methods. This highlights the importance of incorporating the phenotypic context during network inference. Furthermore, an ablation study confirms the synergistic contribution of ChIP-seq, RNA-seq, and phenotypic data, highlighting the value of multimodal integration for accurate phenotype-relevant GRN reconstruction.

## Introduction

In the course of human embryonic development, the first few weeks postconception are critical for establishing the foundation of all tissues and organs. Disruptions during this critical stage can lead to severe pregnancy complications and birth defects. Understanding the regulatory mechanisms and gene expression patterns guiding early human embryogenesis is fundamental for elucidating the origins of various cell types. However, ethical concerns surrounding the manipulation of human embryos and difficulties replicating these processes *in vivo* limit direct investigation. Fortunately, human embryonic stem cells (hESCs), similar to the epiblast stage before gastrulation, can be studied *in vitro* to model early human development due to their self-renewal and pluripotency properties [1]. By analyzing hESCs and their differentiated cell types (Supplementary Figs 1–3) using high-throughput sequencing technologies, researchers can gain deeper insights into the transcriptional mechanisms governing lineage differentiation during human embryonic development.

Gene regulatory networks (GRNs), which represent the interactions between genes and their regulators like transcription factors (TFs), are pivotal for exploring these transcriptional mechanisms. While various methods have been introduced for GRN inference [2–9], most overlook the phenotypic labels of the samples, such as control versus disease status or hESC versus endoderm in this study. Thus, while they can offer insights into general regulatory mechanisms, they cannot identify specific interactions driving phenotypic changes. Recently, we introduced the concept of “phenotype-relevant” GRNs [9], which tries to capture a three-way association between a regulator (e.g. TF), a target gene, and variations in a phenotypic/clinical label. In this study, we introduce InPheRNo-ChIP (Inference of Phenotype-relevant Regulatory Networks with ChIP-seq Integration), a computational framework designed to reconstruct such phenotype-relevant GRNs. This model extends the InPheRNo framework [9] by integrating ChIP-seq, RNA-seq, and phenotypic labels of samples to capture the intricate interplay between TFs, their target genes, and associated phenotypic outcomes. At its core, a probabilistic graphical model (PGM) models the simultaneous effect of multiple TFs on their target genes and the association between the target gene expressions and a phenotypic/clinical variable. By modeling conditional dependences of various summary statistics obtained from different data modalities, it captures the TF–gene and gene–phenotype interactions within the data. Normalization and filtering procedures refine the GRN, enabling confidence scores to be assigned to identified regulatory edges.

We applied InPheRNo-ChIP to reconstruct a phenotype-relevant GRN guiding the differentiation of human embryonic stem cells (hESCs) into definitive endodermal cells (dENs). We showed that compared to alternative methods [2–9], InPheRNo-ChIP can more accurately identify interactions associated with this biological process, based on an experimentally validated network curated from an scRNA-seq CRISPRi study [10]. Additionally, our ablation studies confirmed that each part of InPheRNo-ChIP enhances its accuracy in identifying critical TF–gene interactions for hESC-to-dEN differentiation. Overall, these results suggest InPheRNo-ChIP to be an effective tool in identifying phenotype-relevant GRNs with applications ranging from developmental biology to the study of various diseases [11].

## Materials and methods

### InPheRNo-ChIP pipeline

**Figure 1:**
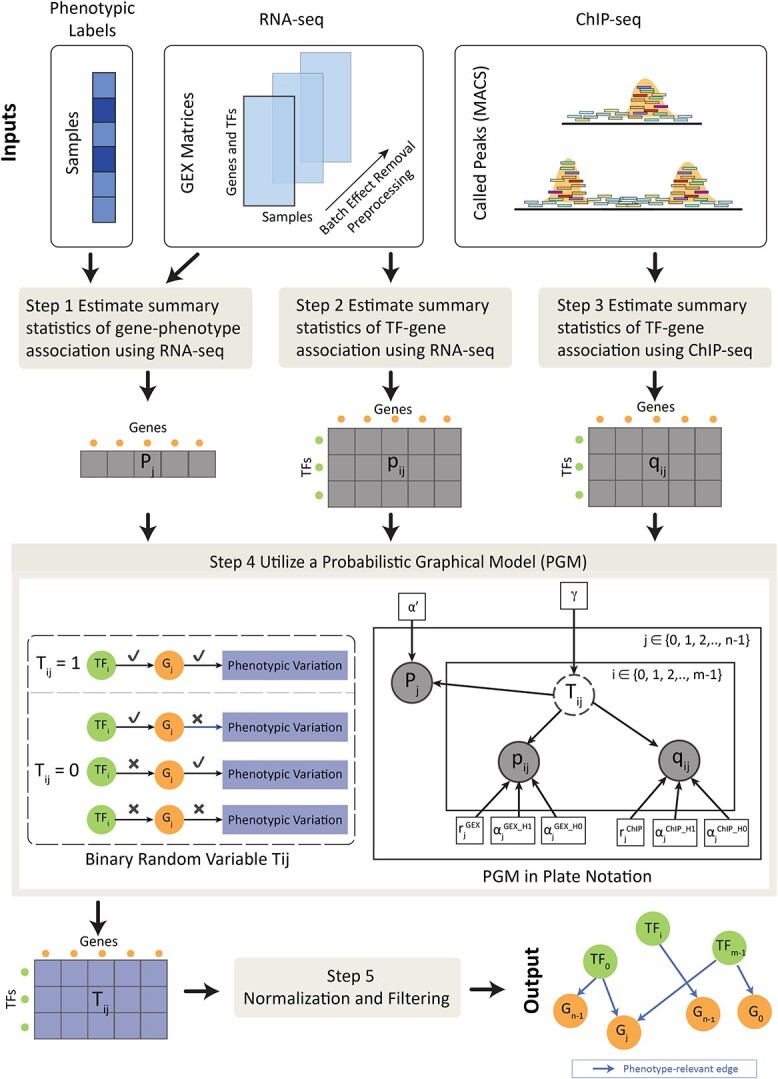
Overview of the InPheRNo-ChIP framework for constructing phenotype-relevant GRNs. First, data cleaning, peak calling, batch effect removal, and other preprocessing steps are applied to the RNA-seq and TF-specific ChIP-seq dataset(s) (detailed in Methods and Supplementary Methods). Then, Step 1 estimates summary statistics for gene–phenotype associations for each gene $\mathrm{j}$ (denoted as ${\mathrm{P}}_{\mathrm{j}}$) using RNA-seq data and phenotype information. Step 2 and Step 3 compute summary statistics for TF–gene associations through RNA-seq (${\mathrm{p}}_{\mathrm{ij}}$s) and ChIP-seq data (${\mathrm{q}}_{\mathrm{ij}}$s), respectively, for a TF $\mathrm{i}$ and gene $\mathrm{j}$ (note that upper-case $\mathrm{P}$ is used for gene–phenotype association summary statistics, while lower-case $\mathrm{p}$ is used for TF–gene association summary statistics using RNA-seq). These summary statistics are then used as observed variables in Step 4, where a PGM calculates posterior probabilities of ${\mathrm{T}}_{\mathrm{ij}}$s. In the PGM plate notation, observed variables are indicated by gray circles, latent variables by white circles, and parameters by squares. The variables *n* and *m* denote the numbers of genes and TFs, respectively. The posterior probabilities for ${\mathrm{T}}_{\mathrm{ij}}$s (output of Step 4), are inputted to Step 5, which employs normalization and filtering procedures to refine the results and reconstruct the final GRN.

InPheRNo-ChIP is a computational tool specifically designed to reconstruct phenotype-relevant GRNs using RNA-seq, TF-specific ChIP-seq, and phenotypic labels (Fig. 1). By “phenotype-relevant,” we refer to interactions that are associated with variations in a phenotypic variable (e.g. responsive versus resistant to a treatment, case versus control, or hESC versus dEN in this study). A carefully designed PGM models the conditional distribution of different random variables including the summary statistics obtained from the input data. The final output is a directed acyclic graph (DAG) representing inferred phenotype-relevant TF–gene regulatory interactions with confidence scores between 0 and 1 (Fig. 1, Step 4).

As input, it receives one (or more) RNA-seq dataset (genes × samples) containing expression profiles of putative target genes and TFs corresponding to a set of samples with known phenotypic labels (e.g. hESC versus dEN) and ChIP-seq data corresponding to DNA–protein interactions of TFs of interest. After data preprocessing, five steps follow to obtain a phenotype-relevant GRN (Fig. 1). The first three steps analyze each data modality to obtain summary statistics capturing gene–phenotype and TF–gene associations. Step 4 uses these summary statistics as observed variables in a PGM and estimates the posterior probability of a set of Boolean random variables, corresponding to each possible TF–gene edge (Fig. 1). A series of normalization and filtering procedures follow to control for different biases and refine this initial graph, ultimately yielding a phenotype-relevant GRN.

#### Step 1: Estimation of gene–phenotype *P*-values using RNA-seq

We integrated three datasets (GEO Accession GSE164361 [12], GSE143371 [13], and GSE160981 [14]) and preprocessed them as detailed in Supplementary Methods. Sample information is provided in Supplementary Table S1. We performed differential expression (DE) analysis using EdgeR [15] to derive *P*-values that capture gene–phenotype associations. The analysis utilized a filtered read count file and a target file specifying each sample’s primary treatment factor (phenotypic labels: “hESC” and “dEN”) and a confounding variable (e.g. Days 0–5, NaNs). We excluded genes previously identified as TFs. We then fitted a negative binomial generalized log-linear model to the normalized counts, incorporating adjustments to control for day-related effects. The *P*-values were then adjusted using the Benjamini–Hochberg false discovery rate (BH-FDR), and we identified 1745 DE genes (BH-FDR < 0.01 and |logFC| > 2) across 28 samples (Supplementary Table S2). Note that the BH-FDR correction was used solely for the initial filtering of the DE gene set; in subsequent analyses, raw *P*-values (denoted as ${P}_j$ for each gene $j$) were used in the PGM since the PGM was designed to model the distribution of raw *P*-values.

#### Step 2: Estimation of transcription factor–gene pseudo *P*-values using RNA-seq

We adopted the approach from our previous study [9] to derive summary statistics of TF–gene associations. We used Limma’s voom function [16] to quantile-normalize the CPM values. Next, we applied a two-step approach, integrating Elastic Net and ordinary least squares (OLS) regression. Each gene’s expression values were inverse quantile–normalized to a standard normal distribution across the 28 samples (Supplementary Table S2) and was used with the Elastic Net regression model. Here, the Elastic Net loss function is described as


$$L = \frac{1}{2n} \| y - Xw \|_2^2 + \alpha \rho \| w \|_1 + \frac{\alpha (1 - \rho)}{2} \| w \|_2^2$$

where $y$ is the expression of a putative target gene across all samples, $X$ is the feature matrix (samples × TFs), $w$ is the vector of coefficients, $\mathrm{\rho} =0.5$, and $\mathrm{\alpha}$ is a hyperparameter that we fine-tuned traversing a regularization path.

For each gene, the TFs with nonzero coefficients were then selected and used in an OLS model to obtain pseudo *P*-values (denoted as ${p}_{ij}$s for each TF $i$ and gene $j$). Note that the *P*-values obtained in this step are termed “pseudo” *P*-values [9] since Elastic Net is first used as a feature selection (prior to fitting the OLS model), biasing the *P*-values toward small values, even under the Null hypothesis (see [9] for more details).

#### Step 3: Estimation of transcription factor–gene pseudo *P*-values using transcription factor ChIP-seq peaks

In this step, we sought to obtain pseudo *P*-values of TF–gene associations based on ChIP-seq data (we used GSE61475). We removed low-quality samples and retained high-confidence peaks using ENCODE’s Irreproducible Discovery Rate (IDR) algorithm [17] and filtered out blacklist regions [18] (see Supplementary Methods). We then applied the T-Gene algorithm (version 5.3.3) from the MEME Suite [19] to derive distance-based *P*-values (denoted as Distance *P*-value) quantifying potential TF–gene associations (denoted as ${q}_{ij}$s for each TF $i$ and gene $j$). T-Gene [19] computes this score by assessing the proximity of each TF ChIP-seq peak to potential target transcription start sites (TSSs). The inputs for this analysis were peak files that met our stringent IDR criteria. We utilized default T-Gene parameters: max-link-distances at 500,000 and max-pvalue at 1.0 and “Homo_sapiens.GRCh38.97.ensembl.transcripts.gtf” as the reference genome. We refined the TF–gene pairs, retaining only those with available ChIP-seq *P*-values for both hESC and dEN. In cases where multiple peaks were present for a single TF–gene pair, we selected the pair with the smallest *P*-value across all peaks. Finally, for any gene, if ${q}_{ij}$ was unavailable but ${p}_{ij}$ existed, we assigned ${q}_{ij}=1$.

#### Step 4: Utilizing a probabilistic graphical model to model conditional probabilities and integrate data modalities

We designed the PGM (the main component of InPheRNo-ChIP) as a DAG (Fig. 1) in which the nodes represent random variables and the edges represent probabilistic dependencies. Central to the PGM are latent Boolean random variables ${T}_{ij}$, which represent the presence or absence of a regulatory relationship between TF $i$ and gene $j$ impacting the phenotype. In words, ${T}_{ij}=1$ implies that TF $i$ regulates gene $j$, thereby influencing the phenotype. Conversely, ${T}_{ij}=0$ implies one of the following: (a) gene $j$ is not associated with the phenotype, (b) TF $i$ does not regulate gene $j$, and (c) both (a) and (b) hold simultaneously. The objective of the phenotype-relevant GRN reconstruction is thus framed as the computation of the posterior probabilities for each ${T}_{ij}$.

The prior distribution of each random variable ${T}_{ij}$ is modeled as a Bernoulli distribution with parameter $\mathrm{\gamma}$ [i.e. ${T}_{ij}\sim \mathrm{Bernoulli}\left(\mathrm{\gamma} \right)$], where $\mathrm{\gamma}$ itself is modeled as a truncated Normal distribution. While we could have simply set $\mathrm{\gamma}$ to a fixed number, we decided that assigning a prior distribution to it provides the model with more flexibility. We set the mean of the underlying (untruncated) Normal distribution of $\mathrm{\gamma}$ to $\mathrm{\mu} =\frac{2}{22}$ and set the truncation boundaries to 0 and $\frac{4}{22}$ (here, 22 is the number of considered TFs in our analysis). This was done to impose sparsity in the network and to introduce the inductive bias that each gene (in average) is regulated by only a few TFs in a phenotype-relevant manner. The parameters of the truncated Normal distribution, however, can be treated as hyperparameters and a user can change them if they require a different inductive bias in the model. The DAG’s topology (Fig. 1) reflects the causal influence of ${T}_{ij}$ on the distributions of the observed variables ${P}_j$ (capturing gene–phenotype association, obtained in Step 1), ${p}_{ij}$ (capturing TF–gene association from RNA-seq, obtained in Step 2), and ${q}_{ij}$ (capturing TF–gene association from ChIP-seq, obtained in Step 3).

The modeling of ${P}_j$ and ${p}_{ij}$ closely follows the methodology established in [9]. Briefly, we use a mixture of Uniform and Beta distributions to model the conditional distribution of ${P}_j$ given its parent nodes (${T}_{ij}$ for $i=1,2,\cdots, {m}_j$) in the DAG. This approach is justified as ${P}_j$ values are “true” *P*-values and follow a Uniform distribution under the null hypothesis. We model them by a Beta distribution under the alternative hypothesis:


$$P_j \mid T_{ij} \sim \begin{cases} \text{ Uniform }(0, 1), & \text{ if } T_{1j} = T_{2j} = \cdots = T_{m_jj} = 0 \\ \text{ Beta }(\alpha{\prime}, 1), & \text{ otherwise }\end{cases}$$

Here, ${m}_j$ is the number of TFs considered as potential regulators of gene $j$, and *α*′ is a parameter between 0 and 1 and controls the degree of bias in the Beta distribution toward smaller values: a smaller value of *α*′ biases the distribution toward values close to zero, while a value close to one turns the Beta distribution into a uniform distribution. We estimate *α*′ by fitting this mixture distribution to a comprehensive set of ${P}_j$ values across all genes, prior to training the PGM.

We model the pseudo *P*-values ${p}_{ij}$using two Beta distributions:


$$p_{ij} \mid T_{ij} \sim\! \begin{cases} \text{Beta }(\alpha_j^{\text{GEX}_\text{H1}}, 1), & \!\!\!\!\!\!\text{ if } T_{ij} = 1 \\ r_j^{\text{GEX }} \text{ Beta }(\alpha_j^{\text{GEX}_\text{H1}}, 1) \!+\! (1 - r_j^{\text{GEX }}) \text{ Beta }(\alpha_j^{\text{GEX}_\text{H0}}, 1), & \!\!\!\!\text{otherwise}\end{cases}$$

where $r_j^{\text{GEX }} \sim \text{ Uniform }(0, 1)$, ${\alpha}_j^{\text{GEX}_\text{H1}}\sim \text{ Uniform } \left(0,0.5\right)$, and $\alpha_j^{\text{GEX}_\text{H0 }} \sim \text{ Uniform }(0.5, 1)$. The reason behind using a Beta distribution, even under the null hypothesis, is that these pseudo *P*-values are biased toward small values, due to the two-step procedure outlined in Step 2. The parameter $r_j^{\text{GEX }}$is a mixing parameter that captures the probability that a TF regulates the gene, but the gene is not associated with the phenotype (see [9] for a detailed discussion).

The random variables ${q}_{ij}$ were obtained using the ChIP-seq data based on the proximity of a TF’s ChIP peak to a gene’s TSS (Step 3). However, since for a single TF, multiple peaks may exist in the proximity of a gene’s TSS, and, since our data included ChIP-seq of both hESC and dEN, multiple *P*-values existed for each TF–gene pair. To consolidate them, we selected the smallest *P*-value to represent ${q}_{ij}$*,* biasing them toward small values; as a result, they should be considered pseudo *P*-values. We modeled the conditional distribution of these random variables as


$$ q_{ij} \mid T_{ij} \sim\!\! \begin{cases} \text{Beta }(\alpha_j^{\text{ChIP}_\text{H1}}, 1), &\!\!\!\!\! \text{ if } T_{ij} = 1 \\ r_j^{\text{ChIP }} \text{ Beta }(\alpha_j^{\text{ChIP}_\text{H1}}, 1)\! +\! (1 - r_j^{\text{ChIP }}) \text{ Beta }(\alpha_j^{\text{ChIP}_\text{H0}}, 1), &\!\! \! \!\!\text{otherwise}\end{cases}$$

where $r_j^{\text{ChIP }} \sim \text{ Uniform }(0, 1)$, $\alpha_j^{\text{ChIP}_{H1}} \sim \text{ Uniform }(0, 0.5)$ and $\alpha_j^{\text{ChIP}_\text{H0}} \sim \text{ Uniform }(0.5, 1)$, in which ${r}_j^\text{ ChIP}$ is a mixing parameter, and ${\alpha}_j^{\text{ChIP}_\text{H0}}$and ${\alpha}_j^{\text{ChIP}_\text{H1}}$ are parameters of the two Beta distributions corresponding to the null and alternative hypotheses, respectively.

InPheRNo-ChIP improves upon InPheRNo by including gene-specific controls that can be used to prune and filter the final GRN (in Step 5). These controls provide thresholds that edges with ${T}_{ij}$ scores below them should be excluded from the final GRN. One of the issues that can be faced by the model is that in the absence of an interaction that is supported by both RNA-seq and ChIP-seq (i.e. ${q}_{ij}$ and ${p}_{ij}$ that are both small), the model may assign high confidence score to an interaction that is only supported by one data modality, which is not desirable. To address this, we added three negative controls for each gene with the intuition that edge scores that are below any of these thresholds should be excluded from the final GRN. We consider these negative controls as random variable nodes corresponding to an edge between an artificial TF and the gene of interest, whose ${q}_{ij}$ and ${p}_{ij}$ are selected accoding to Table 1: the pseudo *P*-value corresponding the data modality not supporting the edge is set to 1, while the other pseudo *P*-value is set to the smallest observed value for that gene (based on actual TFs).

**Table 1:** Control variables added to the PGM to ensure that each edge is supported by all data modalities.

Controls for each gene $j$	Pseudo *P*-value of TF–gene association from RNA-seq	*P*-value of TF–gene association from ChIP-seq	*P*-value of gene–phenotype association
ref_PIE	1	${q}_{ij}^{\ast }$	${P}_j$
ref_Q	${p}_{ij}^{\ast }$	1	${P}_j$
ref_PIE_Q	1	1	${P}_j$

In this table, $i$ indexes different TFs, while $j$ is the index of the gene. In this table, ${P}_j$ is the observed gene–phenotype *P*-value of the gene of interest, ${q}_{ij}^{\ast }$ is the smallest observed ${q}_{ij}$ among all TFs for the fixed gene $j$, and ${p}_{ij}^{\ast }$ is the smallest observed ${p}_{ij}$ among all TFs for the fixed gene $j$.

Posterior probabilities for all these Boolean variables are then estimated via the Markov chain Monte Carlo (MCMC) sampling [20, 21] and used to construct an initial GRN. We used PyMC version 5.6.1, leveraging PyTensor for computational efficiency [22]. Table 2 summarizes key parameters used for obtaining these posteriors. We ran multiple chains with distinct initializations to avoid local minima. Each chain has a total of ${N}_i+{N}_t$ iterations, discarding the first ${N}_t$ tuned samples.

**Table 2:** Summary of input arguments for the pm.sample() function in PyMC5.

Argument	Description
${N}_b$	Number of iterations to burn, set to 400 (10% of the of the total iterations, ${N}_i$).
${N}_c$	Number of MCMC traces, set to 4.
${N}_i$	Number of samples/iterations to be drawn, set to 4000.
${N}_t$	Number of tuning/burn-in samples to be discarded, set to 2000.

#### Step 5: Refinement of the phenotype-relevant gene regulatory network

In Step 5, InPheRNo-ChIP processes gene batches deserialized from the PyMC5 “MultiTrace” object, discarding initial burn-in samples to enhance reliability. This step produces a tensor of posterior probabilities ${T}_{ij}$s (MCMC traces × genes × TFs). We compute the mean posterior probabilities across MCMC traces for each gene–TF pair. To further refine these results, we first min-max normalize the mean posterior probabilities for gene $j$across all TFs and artificial controls (to ensure that the scores are comparable across different genes). The normalized scores are then sorted for each gene in descending order​. Next, for each gene, we identify the index where there is a large drop in the consecutive scores (forming the first local maximum). Scores below the corresponding threshold will not be included in the final GRN (referred to as Filter1). Next, we use the three negative controls discussed in Table 1 and exclude any interactions that are scored lower than any of them (referred to as Filter2). Finally, scores that are smaller than the parameter $\mathrm{\mu}$ of the truncated Normal distribution (set to 2/22 in this study) are excluded (referred to as Filter3).

### Alternative gene regulatory network inference methods

To evaluate the performance of InPheRNo-ChIP, we conducted a comparative analysis against 10 GRN inference methods: GENIE3 and GRNBoost2 from Arboreto package [2], Inferelator 3.0 [3], PANDA [4], NetREX [5], MERLIN+Prior and its variant PGG+Prior [6], KBoost [7], iRafNet [8], and InPheRNo [9]. We selected these based on their widespread use and their ability to integrate multiple data modalities. We largely adhered to the recommended parameter settings from the original authors, with minor adjustments necessary for this study (Supplementary Methods). Of these methods, three could not utilize ChIP-seq data (InPheRNo, GENIE3, GRNBoost2). For the rest of the algorithms, we utilized both RNA-seq and ChIP-seq data. Moreover, to incorporate the phenotypic labels, we ran each method based on differential network analysis (DiNA) and context-specific network analysis (CsNA), detailed in Supplementary Methods and Supplementary Fig. S2.

## Results

### InPheRNo-ChIP enables phenotype-relevant gene regulatory network inference using multimodal data

InPheRNo-ChIP is a computational pipeline based on a PGM that integrates TF-specific ChIP-seq, RNA-seq, and phenotypic/clinical labels to identify “phenotype-relevant” GRNs [9]. The aim of this model is to go beyond finding TF–gene regulatory interactions but instead pinpoint interactions that are associated with variations in a phenotypic label (or outcome). This model incorporates summary statistics derived from gene expression, DNA–protein interactions, and sample-level phenotypic labels and systematically models them as observed random variables within a PGM. The PGM is designed to navigate the intricate challenges of integrating independent datasets and diverse data types while correcting for potential biases to reconstruct GRNs that are relevant to phenotypic variations (Fig. 1 and Methods).

The InPheRNo-ChIP’s pipeline is structured around five steps. In the first step, differential expression analysis is performed to calculate summary statistics (*P*-values) capturing the relationship between the expression profile of genes and phenotypic labels. In the second step, regression models are used to estimate pseudo *P*-values relating the expression of genes to TFs, similar to the procedure described in Emad and Sinha [9]. In the third step, the ChIP-seq binding peaks are analyzed using T-Gene [19] to obtain *P*-values capturing TF–gene interactions based on the distribution of the ChIP peaks. In the fourth step, these three sets of summary statistics are provided as observed variables in a carefully designed PGM that models the conditional distribution of different random variables. In this PGM, for each TF–gene pair, a Boolean random variable ${T}_{ij}$ is defined that if equal to 1 (or “true”) implies that the $i$*-*th TF regulates the $j$*-*th gene in a phenotype-relevant manner (capturing TF–gene and gene–phenotype relationships). Posterior probabilities for these Boolean variables are estimated via MCMC sampling [20, 21] and used to construct an initial regulatory graph capturing interactions among TFs, genes, and the phenotype. The fifth step involves a series of normalization and filtering procedures to control for different biases and refine this initial graph, ultimately yielding a phenotype-relevant GRN (Fig. 1, Methods).

Compared to its predecessor, InPheRNo [9], InPheRNo-ChIP provides several key improvements. First, while InPheRNo uses only gene expression data (and phenotypic labels), InPheRNo-ChIP expands its scope by integrating both gene expression and ChIP-seq datasets. This multimodal approach provides a more comprehensive understanding of underlying regulatory mechanisms. Second, in contrast to InPheRNo’s one-size-fits-all global threshold for graph pruning, InPheRNo-ChIP adopts a more targeted strategy to refine the initial draft GRN into a final phenotype-relevant GRN. For each gene, the framework performs several normalization and filtering steps to identify the most probable regulatory interactions for that gene. Third, the framework has transitioned from PyMC version 2 and Python 3.6 to PyMC version 5 [13] and Python 3.11. This shift not only optimizes the parallelism in MCMC sampling (which improves the computational efficiency and enhances the convergence diagnostics) but also ensures long-term reproducibility.

### InPheRNo-ChIP identifies lineage-relevant gene regulatory networks in human embryonic stem cell differentiation

We first removed the batch effect present in the RNA-seq data (since we used three independent studies) using ComBat-seq [23] (Supplementary Methods and Supplementary Fig. S3A). The final dataset comprised *n* = 28 samples, including hESC (*n* = 11) and dEN (*n* = 17) cell types. We performed DE analysis on non-TF genes, identifying 968 downregulated and 777 upregulated genes in dEN compared to hESC (|logFC| > 2 and FDR < 0.01) (Supplementary Fig. 3B). These genes and their associated *P*-values served as the observed data for modeling gene–phenotype relationships within the InPheRNo-ChIP pipeline (see Supplementary Table S2 for the complete list). Separately, we processed TF ChIP-seq peaks (GSE61475 [24]) with T-Gene [14] to obtain a second set of pseudo *P*-values reflecting TF–gene regulatory relationships. We focused on 22 TFs for which ChIP-seq data were available in both hESC and dEN cell types. To derive statistical evidence of TF–gene regulatory interactions (Fig. 1), an Elastic Net regression model was employed to model the expression of each differentially expressed gene as a linear function of the expression values of the 22 TFs. Subsequent OLS regression yielded pseudo *P*-values representing potential TF–gene associations.

**Figure 2:**
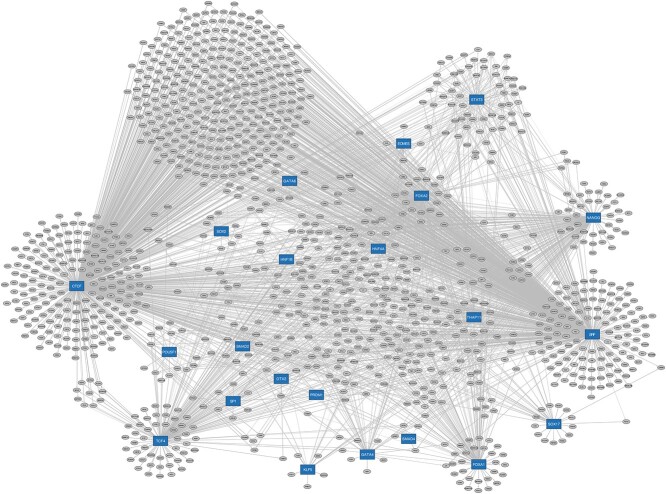
Phenotype-relevant GRN underlying hESC to dEN differentiation. This figure displays the inferred GRN that is relevant to hESC-to-dEN differentiation, visualized using Cytoscape v3.10.2 [25]. In the figure, blue rectangles represent TFs with available ChIP-seq data, and gray circles denote differentially expressed (DE) genes. Directed edges, whose thickness is proportional to the InPheRNo-ChIP confidence score (0–1 scale), connect TFs to their target genes, illustrating the strength of each regulatory interaction. The network data used to generate this figure is provided in Supplementary Table S3, in the “RnCnP” tab.

After these steps, for each gene, we ended up with three sets of summary statistics: one set representing gene–phenotype associations and two for TF–gene interactions from RNA-seq and ChIP-seq data. We only kept differentially expressed genes that were also present in the ChIP-seq data and had at least one significant TF–gene association as evidenced by *P*-values from either T-Gene or Elastic Net analyses. This process yielded 1240 final genes, whose summary statistics were used as input to InPheRNo-ChIP’s PGM. After estimating the posterior probabilities and the normalization and filtering steps, we obtained a final GRN reported in Supplementary Table S3 (Sheet 2, denoted as “RnCnP”) and Fig. 2.

Table 3 presents each TF, sorted by the number of genes they regulate in the final GRN, where the average target gene percentage across the 22 TFs equals 8.76%. *CTCF* had the largest number of target genes. In addition to its well-characterized insulator function, CTCF is widespread at regulatory elements and mediates promoter–distal enhancer interaction across development [24]. This TF is crucial for hESC proliferation and general cellular viability, so its ubiquity is not surprising [26–28]. Nanog is a well-established pluripotency master-regulator [29], while TCF4 typically interacts with β-catenin to mediate Wnt signaling effects, which can influence various cellular processes including proliferation, differentiation, and migration [30, 31]. FOXA1/2, GATA4, and SOX17 execute critical transcriptional programs essential for endoderm development and its derivative organs [32–34]. These TFs do not work in isolation but often collaborate with signaling pathways such as Wnt and TGF-beta, executing complex tasks during embryogenesis and organogenesis [35].

**Table 3:** List of the TFs and the number of their inferred target genes.

TFs	Num. target genes	Percentage (%)
*CTCF*	733	59.11
*SRF*	686	55.32
*TCF4*	193	15.56
*NANOG*	187	15.08
*FOXA1*	114	9.19
*FOXA2*	108	8.71
*STAT3*	59	4.76
*THAP11*	53	4.27
*SOX17*	42	3.39
*GATA4*	42	3.39
*HNF4A*	27	2.18
*SP1*	26	2.1
*EOMES*	25	2.02
*KLF5*	22	1.77
*POU5F1*	19	1.53
*GATA6*	12	0.97
*OTX2*	11	0.89
*SMAD2*	11	0.89
*SOX2*	10	0.81
*SMAD4*	4	0.32
*HNF1B*	4	0.32
*PRDM1*	3	0.24

The percentages are calculated as the number of inferred target genes regulated by each TF in a phenotype-relevant manner divided by *n* = 1240.

### InPheRNo-ChIP outperforms alternative methods

Since InPheRNo-ChIP seeks to find “phenotype-relevant” regulatory edges (instead of global regulatory interactions), finding ground-truth GRNs to systematically evaluate its performance was challenging: synthetic data generated by simulators [36–39] or curated databases gathering interactions from various experimental studies and contexts are not suitable due to their lack of context-specificity and phenotypic relevance. Instead, we turned to a single-cell RNA-sequencing-based CRISPRi screening study [10] to reconstruct ground truth networks essential for early human endoderm formation. This study began with atacTFAP analysis to identify candidate TFs actively involved during the hESC-to-dEN differentiation process. Subsequently, scRNA-seq-based CRISPRi screening revealed four distinct phenotypic clusters, each representing a different stage of endoderm differentiation. These data offer a robust and contextually relevant framework for evaluating the ability of InPheRNo-ChIP and other GRN inference methods to find phenotype-relevant GRNs. Based on this study, we reconstructed three ground truth networks: *FOXA2*-oriented network, *SMAD2*-oriented network, and *SOX17*-oriented network. The details of how these networks were established are provided in the Supplementary Methods.

Since most GRN inference methods do not readily incorporate phenotypic information, we used two strategies with each method: context-specific network analysis (CsNA) and differential network analysis (DiNA) (Supplementary Fig. S2). In CsNA, (e.g. [40]), differentially expressed genes are first identified, and a GRN inference method is applied to link those genes to their regulating TFs. In DiNA, two GRNs are constructed corresponding to each phenotypic label (separately), and edges present in one but not the other are reported as the output [41] (see Supplementary Methods for details).

We evaluated the performance of our model against 10 GRN inference methods capturing a wide range of strategies, including InPheRNo [9], GENIE3 and GRNBoost2 from Arboreto package [2], Inferelator 3.0 [3], PANDA [4], NetREX [5], MERLIN+Prior and its variant PGG+Prior [6], KBoost [7] and iRafNet [8]. The first three use only RNA-seq, while we ran the rest of the methods integrating both RNA-seq and ChIP-seq data (see Supplementary Methods for details). Except for InPheRNo, which has a built-in strategy to use phenotypic labels, we ran the rest of the methods using both CsNA and DiNA strategies. The evaluation was performed based on the ground-truth GRN of each TF separately (henceforth “TF-level”) and based on the aggregated network of all three TFs collectively (henceforth “edge-level”). Figs 4 and 5 show the performance of InPheRNo-ChIP (denoted as “InPheC RnCnP”), two ablated versions of it (“InPheC RnP” and “InPheC CnP”), as well as various alternative GRN inference methods ran in different configurations [the details of these configurations are provided in Supplementary Table S4 (Sheet 4)]. These figures show the ratio of area under the precision–recall curve (AUPRC) compared to that of a random predictor, as the main metric for evaluation since it is appropriate for evaluation in sparse setups and is commonly used in evaluating GRN inference methods [36]. Additionally, Supplementary Table S4 contains various other metrics such as the Matthews correlation coefficient (MCC), area under the receiver operating characteristic (AUROC) curve, precision at *k*, and F-score.

**Figure 3:**
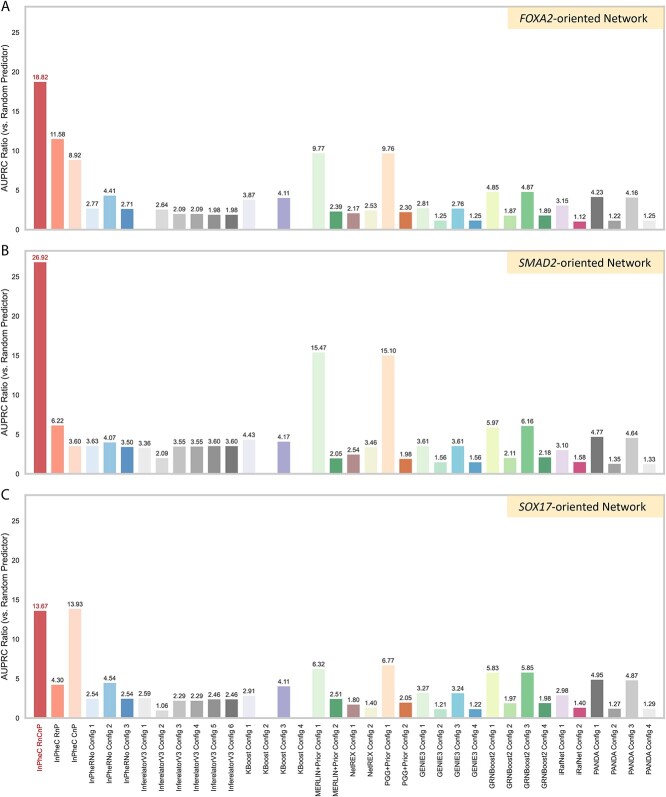
Performance comparison of GRN inference algorithms for the interactions of different TFs. Three TF-level networks were reconstructed from a single-cell RNA-sequencing-based CRISPRi screening study [10]. Each network focuses on a specific TF and its experimentally validated target genes. Bar plots illustrate the AUPRC ratio relative to a random predictor. Panels depict results within the (A) *FOXA2*-oriented, (B) *SMAD2*-oriented, and (C) *SOX17*-oriented networks. Algorithms’ names were standardized for consistent abbreviations across all TF networks. A configuration number appended to the abbreviation signifies different configurations or setups of the algorithms. Details of each configuration, including the data modalities used (RNA-seq, ChIP-seq, or both) and specific parameter settings, are provided in Supplementary Table S4, within the “Configs” tab. For example, “MERLIN+Prior variant 1” corresponds to the “MerlinP-Score (CsNA)” configuration detailed in Supplementary Table S4. Note that within the “InPheC” family (the method developed in this study), RnCnP represents the complete model (i.e. InPheRno-ChIP), while the other two bars represent its ablated variants (“InPheC RnP” does not utilize ChIP-seq data, while “InPheC CnP” does not utilize RNA-seq data). The absence of a bar for a specific algorithm indicates a failure to detect meaningful edges within the ground truth network, leading to an undefined (NaN) AUPRC score.

**Figure 4:**
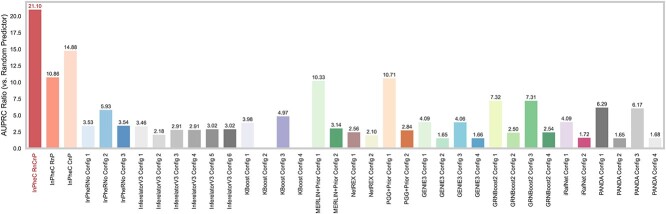
Performance of different GRN inference methods using the edge-level evaluation. This bar graph displays the performance of various GRN inference methods, comparing each method’s inferred network against a ground-truth edge-level network. This ground truth network was constructed from all three TF-level networks derived from a scRNA-seq CRISPRi study. The height of each bar illustrates the ratio of the AUPRC to that of a random predictor. Details of the configurations for each algorithm, including data modalities used and parameter settings, can be found in Supplementary Table S4, within the “Configs” tab.

In the *FOXA2-*oriented network, InPheRNo-ChIP with both RNA-seq and ChIP-seq data achieves an AUPRC of 0.41 and an AUPRC ratio (compared to random) of 18.82 (Fig. 3A), outperforming all the other models with a large margin, followed by its ablated variations and MERLIN+Prior and PGG+Prior. A comprehensive assessment (Supplementary Table S4) reveals that algorithms have distinct strengths: while Inferelator 3.0 has a configuration that achieves an AUROC of 0.76, InPheRNo-ChIP maintains advantageous for sparse GRN analysis due to its superior AUPRC ratio. In the *SMAD2*-oriented network, InPheRNo-ChIP once again outperforms all other models, achieving an AUPRC ratio of 26.92. PGG+Prior Config 1 and MERLIN+Prior Config 1 achieve AUROCs of 0.796 and 0.79, respectively, which are higher than the 0.75 achieved by InPheRNo-ChIP. These two configurations also show relatively high AUPRC ratios of 15.47 and 15.10. In contrast, other methods, including the ablated variants of our model InPheC-RnP and InPheC-CnP, demonstrate AUPRC ratios below 7 (see Supplementary Table S4). In the *SOX17*-oriented network, InPheRNo-ChIP outperforms other models in terms of the AUPRC ratio (Fig. 3C). One configuration of PANDA (Config 1) achieves the highest MCC score of 0.39 and an AUROC score of 0.68; however, it falls short in terms of the AUPRC ratio (achieving a ratio of 4.94). Conversely, two of GRNBoost2 configurations (Configs 1 and 3) demonstrate balanced performance across metrics, each registering the highest AUROC score of 0.69 and an AUPRC ratio of ~5.8. The observations discussed above in TF-level evaluation were also supported when considering all interactions simultaneously in the edge-level evolution (Fig. 4), revealing that InPheRNo-ChIP with all data modalities (InPheC-RnCnP) outperforms alternative models.

### Integration of multi-omics data improves the performance of InPheRNo-ChIP

Next, we compared the performance of InPheRNo-ChIP when using both ChIP-seq and RNA-seq data (InPheC-RnCnP) against its performance when only RNA-seq data or only ChIP-seq data are used to capture TF–gene associations (InPheC-RnP and InPheC-CnP, respectively). In the *FOXA2*-oriented network, the AUPRC ratios of InPheC-RnP and InPheC-CnP were 11.58 and 8.92, respectively, compared to 18.82 for InPheC-RnCnP. In the *SMAD2-*oriented network, InPheC-RnCnP achieved an AUPRC ratio of 26.92, greatly surpassing both InPheC-RnP (AUPRC ratio = 6.22) and InPheC-CnP (AUPRC ratio = 3.60), highlighting the importance of incorporating both RNA-seq and ChIP-seq data (Fig. 3B). Interestingly, in the *SOX17*-oriented network, InPheC-CnP outperformed InPheC-RnCnP by a small margin; however, when considering all TFs collectively in the edge-level evaluation (Fig. 4), InPheC-RnCnP has the best performance, signifying the importance of integrating these two data modalities.

Another interesting observation is the better performance of InPheC-RnP compared to InPheRNo [9], the previous version of our model. Even though both of these models only use RNA-seq and phenotypic labels, InPheC-RnP relies on various *post hoc* normalization and filtering steps to improve its predictions, which is signified by its superior performance compared to InPheRNo.

### Enrichr gene set enrichment analysis reveals endoderm-derived tissues

**Figure 5:**
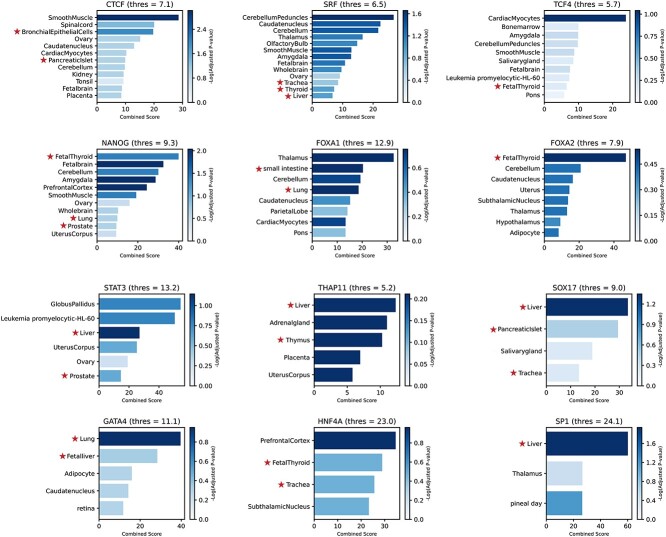
Gene set enrichment analysis results for the top 12 TFs identified by InPheRNo-ChIP, focusing on target genes with confidence scores >0.5. Each subplot displays terms with a combined score above the 85th percentile threshold of each TF (indicated in brackets). The intensity of the shading represents the −log10 of the adjusted *P*-value, with higher intensities indicating stronger statistical significance. Endoderm-derived tissues are marked with a star. Details of the complete list can be found in Supplementary Table S5.

To elucidate the regulatory dynamics of gene expression governing endoderm development, we performed gene set enrichment analysis using the Enrichr platform [42] on the top 12 TFs and their target genes with confidence score >0.5, identified by InPheRNo-ChIP. The analysis (Fig. 5) revealed significant enrichment of endoderm-derived tissues, notably liver, lung, pancreas, and thyroid [43, 44]. Particularly, endodermal TFs such as *GATA4*, *FOXA1/2*, and *SOX17* showed strong associations with these tissues, underscoring their pivotal roles in the regulation of key developmental pathways. We also found that some endoderm-related TFs, particularly *FOXA1* and *FOXA2*, showed association with various brain tissues, consistent with their importance in dopaminergic neurons and their presence in other neural tissues [45–47].

## Discussion

GRN inference is essential for understanding the regulatory mechanisms underlying cellular functions and developmental processes, especially during human embryogenesis. While most GRN inference methods focus on specific biological contexts and often need additional analyses, such as the DiNA strategy, to identify phenotype-relevant interactions, InPheRNo [9] stands out by incorporating phenotypic information directly into the modeling process. However, its application is limited as it only integrates transcriptomic data and cannot incorporate other data types.

In this study, we developed InPheRNo-ChIP, an advanced version of InPheRNo [9], and applied it to identify regulatory interactions that are relevant in the hESC-to-dEN differentiation. InPheRNo-ChIP employs a PGM at its core, which facilitates the integration of TF–gene associations from both RNA-seq and ChIP-seq data, as well as gene–phenotype associations. Furthermore, it considers the simultaneous effects of multiple TFs on each gene, thus providing a nuanced understanding of regulatory mechanisms. Advancing beyond InPheRNo, InPheRNo-ChIP integrates ChIP-seq data via modeling of summary statistics, which provides direct evidence of TF binding to DNA. Furthermore, InPheRNo-ChIP implements advanced fine-grained thresholding and filtering techniques, crucial for pinpointing TF–gene connections that are particularly relevant to the differentiation process of interest.

After identifying GRNs relevant to the hESC-to-dEN differentiation process, we observed that TFs with a high percentage of phenotype-relevant target genes are pivotal in this process. Benchmarking against ground truth networks derived from an scRNA-seq CRISPRi study, which identified regulatory interactions and their functional significance for *FOXA2*, *SMAD2*, and *SOX17* in a phenotype-specific manner during endoderm differentiation, clearly showed InPheRNo-ChIP’s effectiveness in pinpointing such regulatory mechanisms. Specifically, despite employing DiNA and CsNA strategies for other GRN inference methods to incorporate phenotype information as well as including both ChIP-seq and RNA-seq in various configurations, InPheRNo-ChIP achieved superior performance, providing evidence in support of the effectiveness of its PGM’s strategy of simultaneously modeling TF–gene and gene–phenotype associations. An ablation study further confirmed the synergistic contribution of multimodal data integration, underlining the value of using both ChIP-seq and RNA-seq datasets. Enrichr analysis of target genes identified by InPheRNo-ChIP showed significant enrichment in endoderm-derived tissues like liver, lung, pancreas, and thyroid, confirming the biological relevance of our findings. Collectively, these results showed that InPheRNo-ChIP can be used as a robust tool for constructing biologically relevant GRN networks, particularly in the context of developmental biology.

There are several limitations that we should point out. First, InPheRNo-ChIP relies on the availability of TF-specific ChIP-seq, which is not always available. In fact, the availability of noncancer ChIP-seq data restricted our analysis to only 22 TFs. Second, evaluating phenotype-relevant regulatory edges (as opposed to global edges) is quite challenging. While various simulators have been developed that enable the generation of molecular data while imposing a known GRN [36–39], they do not enable the imposition of phenotype-relevant edges. Similarly, typical gold-standard curated GRNs that are widely used for evaluation are not appropriate for this context, as they do not correspond to phenotype-relevant edges. Due to these challenges, we had to rely on a scRNA-seq CRISPRi study to provide ground truth data. However, from this study, we were only able to construct three TF-oriented networks, which largely limits our ability to comprehensively validate the full spectrum of regulatory interactions inferred by InPheRNo-ChIP. Another limitation is the fact that the Elastic Net algorithm used in this study for obtaining summary statistics from RNA-seq is a linear model and does not capture nonlinear associations between TF and genes. While using nonlinear alternatives is possible, the main challenge is developing an efficient method for obtaining summary statistics from them. Finally, the current version of InPheRNo-ChIP does not utilize other important data modalities such as chromatin accessibility or genomic information, and such data types (if available) should be used as preprocessing steps to narrow down the list of relevant TFs and genes (an approach that is commonly used in the literature). It would be interesting, however, to obtain and directly incorporate summary statistics from these data modalities in the PGM for more accurate GRN inference in the future.

**Key Points**

- We introduce InPheRNo-ChIP, a novel computational framework that integrates ChIP-seq and RNA-seq data with phenotypic information to infer phenotype-relevant gene regulatory networks (GRNs).
- InPheRNo-ChIP uses probabilistic graphical models and detailed gene-wise normalization and filtering to enhance the accuracy of GRN reconstruction.
- This study applies InPheRNo-ChIP to infer a GRN that is relevant to the differentiation of hESC to dEN.
- The effectiveness of InPheRNo-ChIP is rigorously tested against 10 established GRN inference methods using experimental ground truth networks derived from a scRNA-seq CRISPRi study, showing great performance in identifying phenotype-relevant regulatory interactions.

## Supplementary Material

SupplementaryFileS1_bbae592

TableS1_bbae592

TableS2_bbae592

TableS3_bbae592

TableS4_bbae592

TableS5_bbae592

## Data Availability

All data analyzed in this study are publicly available from the GEO repository (see Supplementary Table S1 for sample information). Our implementation of the InPheRNo-ChIP pipeline is open-source and available on GitHub at https://github.com/Emad-COMBINE-lab/InPheRNo-ChIP.
