# Supplementary material for: Deciphering lineage-relevant gene regulatory networks during endoderm formation by InPheRNo-ChIP
Source: Brief Bioinform. 2024 Nov 13;25(6):bbae592. doi: 10.1093/bib/bbae592 (PMC11558691; doi:10.1093/bib/bbae592)
Supplement: SupplementaryFileS1_bbae592 [file supplementaryfiles1_bbae592.pdf]

Supplementary Methods

RNA-seq and ChIP-seq Data Acquisition

The RNA-seq data were obtained from the GEO repository corresponding to accession numbers GSE164361, GSE143371, and GSE160981. Additionally, we obtained ChIP-seq data from GEO under the accession code GSE61475, focusing exclusively on samples from hESC and dEN lineages. For a comprehensive list of samples see Supplementary Table S1. We identified 22 TFs within these datasets (based on the list of human TFs from AnimalTFDB 3.0 [1]) with both RNA-seq and ChIP-seq data (both for dEN and hESC lineages). After finding the intersection of the genes present in these datasets and removing genes located on chromosomes X and Y to minimize the potential confounding effects of sex-specific gene expression, we ended up with a list of 19,175 protein-coding genes and n = 28 samples (either hESC or dEN).

We also acquired the ENCODE blacklist [2], a curated list of genomic regions known for exhibiting aberrant or elevated signals in next-generation sequencing (NGS) experiments, irrespective of the cellular context. This list was obtained from the Boyle Lab GitHub repository (https://github.com/Boyle-Lab/Blacklist), specifically the file 'hg19-blacklist.v2.bed', and it identified 834 regions designated as artifacts, which we excluded from our analysis.

RNA-seq data preprocessing

Due to the limited sample sizes often found in individual RNA-seq studies relevant to hESC differentiation, we decided to download and integrate data from three publicly available RNA-seq studies to derive TF-gene and gene-phenotype association p-values. We obtained raw gene-level counts from the GEO repository (accession numbers: GSE164361, GSE143371, and GSE160981). To ensure our analysis focused specifically on the hESC-to-dEN differentiation process, we only kept gene expression profiles that are important for this process. Out of a total of 200 raw samples across these datasets, 28 met our inclusion criteria: twelve from GSE164361, ten from GSE143371, and six from GSE160981 (Supplementary Table S1). We then standardized gene IDs to HGNC symbols and excluded genes from chromosomes X and Y to eliminate any sex-specific biases not relevant to the general process of endoderm formation. To further refine the dataset, we applied a filtering criterion to remove low-expressed genes. More specifically, using the EdgeR pipeline [3], we calculated the Counts Per Million (CPM) for each gene across all samples. We then retained only those genes for which the CPM exceeded a threshold of one in at least two samples. Since these datasets were obtained from three independent studies and we observed a study-specific bias (Supplementary Figure S3), we applied the ComBat-seq package [4] to remove this bias (akin to batch effect).

The aggregated RNA-seq dataset subsequently serves as input for Steps 1 and 2 of the InPheRNo-ChIP process.

ChIP-seq data preprocessing

To ensure the quality and relevance of our ChIP-seq analyses for the study, we started by obtaining BED files from the GEO dataset GSE61475. Initial data cleaning involved removing low-quality samples and those with annotations such as “dEN_shGATA4” or “chip-antibody with NA,”

as well as correcting errors in sample names and standardizing cell type annotations. This careful selection process left us with 22 TFs with high-quality BED files for both hESC and dEN lineages.

We then applied the ENCODE's Irreproducible Discovery Rate (IDR) algorithm [5] to carefully assess peak reproducibility across biological replicates. Given the variable number of replicates for each TF within a lineage, such as dEN, our approach stratified samples into three categories: (1) samples with a single replicate were included in subsequent analyses without IDR assessment due to the requirement of two replicates for IDR; (2) samples with two replicates underwent pairwise IDR comparisons; (3) samples with more than two replicates underwent all possible pairwise IDR comparisons. In all cases involving multiple replicates (i.e., categories (2) and (3)), we retained the replicate pair exhibiting the highest number of peaks exceeding the stringent IDR threshold (0.05). These high-confidence peaks were used for further analyses.

Lastly, we removed known blacklist regions [2], to minimize the impact of potential artifacts. To accomplish this, we employed BEDTools [6], specifically its intersect() function with the '-v' flag, to systematically exclude regions listed in the ENCODE blacklist and the NCBI issue mapping list from our dataset.

These refined datasets serve as inputs for Step 3 of the InPheRNo-ChIP pipeline.

Benchmarking Against Alternative GRN Inference Methods

Incorporating phenotypic lables with baseline GRN inference methods using DiNA and CsNA

Two common approaches for incorporating binary phenotypic labels in GRN inference includes Differential Network Analysis (DiNA) and Context-specific Network Analysis (CsNA) (Supplementary Figure S2). We used DiNA and CsNA strategies for all GRN inference methods that do not utilize phenotypic information to allow a fair comparison.

In DiNA, using any GRN inference method, two separate GRNs were constructed: one for hESC (using only hESC samples) and one for dEN (using only dEN samples). Then, for each edge (between a TF and a gene), the absolute difference between the edge weight in the dEN and hESC GRNs were calculated to represent the edge weight of the final GRN (used for evaluation).

In CsNA (which in principle is more similar to the approach of InPheRNo and InPheRNo-ChIP in incorporating the phenotypic information than DiNA), first a set of differentially expressed genes (DEGs) were identified; then, a GRN was constructed only for DEGs, but using both dEN and hESC samples (simultaneously) to form a single GRN. Although CsNA has similarities with our approach, unlike our models, it does not utilize the strength of association between gene and phenotype.

Input Data Configuration of baseline GRN inference methods

The foundational setup for our analysis includes RNA-seq data for all methods, with ChIP-seq data additionally provided to algorithms capable of incorporating prior network information.

For the RNA-seq gene expression (GEX) data, we performed the same preliminary data preprocessing and applied voom quartile transformation as described in InPheRNo-ChIP Step 2. The result was three gene-by-sample matrices: GEX_{dEN}, which contains only samples from the dEN lineage; GEX_{h64}, comprised solely of samples from the hESC lineage; and GEX_{mix}, which contains samples from both lineages. The lineage-specific matrices, GEX_{dEN} and GEX_{h64}, are prepared for DiNA strategy, while GEX_{mix} is prepared for the CsNA strategy.

For the prior networks, we utilized the output from the T-Gene algorithm in Step 3 of InPheRNo-ChIP, which provided putative genes and their corresponding Distance p-values in the dEN and hESC lineages separately. When multiple p-values for a single TF-gene pair were available within a lineage, we selected the smallest p-value. These values were then used to create binarized data matrices: $Prior_{dEN}$ matrix for the dEN lineage and $Prior_{h64}$ matrix for the hESC lineage, both specifically prepared for use in the DiNA strategy. P-values were transformed into binary scores by setting a threshold: values below a predefined cut-off of 0.05 were assigned a binary score of 1, indicating significant interaction, and all others were set to 0. For CsNA strategy, we created a third matrix, $Prior_{mix}$, by combining the p-values from both lineages for each TF-gene pair. When multiple peaks were present across lineages for a single TF-gene pair, we selected the smallest p-value from all available peaks to represent the interaction. This value was then binarized using the same threshold criterion as the other two matrices. All three matrices are formatted as Gene x TF, with cell values set to either 1 or 0 to indicate the presence or absence of a regulatory interaction.
Additionally, for algorithms that require a list of TFs as input, we created a TF list file. This file includes the names of all 22 TFs under study and is used to specify the regulators considered in the GRN inference.

This baseline configuration serves as the standard input for all evaluated GRN inference methods. Depending on the specific requirements of each method, modifications may be applied to these data sets to suit different algorithmic needs, as detailed in the subsequent sections.

Details of GRN inference methods
In this study, we extensively benchmarked ten GRN inference tools to evaluate their effectiveness in modeling complex regulatory networks. Among these tools, GENIE3 and GRNBoost2 [7] are exclusively based on gene expression data. GENIE3 uses a random forest algorithm to evaluate the regulatory potential of each gene, whereas GRNBoost2 employs gradient boosting, optimized for processing large datasets efficiently. iRafNet [8] enables multi-omics data integration by introducing a weighted sampling scheme under GENIE3 framework. InPheRNo [9] and MERLIN+Prior [10] leverage probabilistic graphical models (PGMs) to infer GRNs, yet each focus on distinct aspects of the network. InPheRNo directly integrates phenotype information into its GRN inference, effectively linking gene regulation to phenotypic outcomes. In contrast, MERLIN+Prior employs PGMs to address gene-specific regulatory actions and modular interactions, thereby facilitating the integration of multi-omics data into the GRN construction. Inferelator 3.0 [11] and NetREX [12] both utilize Network Component Analysis (NCA) but approach GRN modeling differently. Inferelator 3.0 [11] uses NCA to estimate TF activities from existing gene expression data and a prior network, using these activities to predict regulatory

relationships. NetREX [12] adopts a dynamic methodology by actively modifying the prior network—adding and removing edges—based on its alignment with new gene expression data. PANDA [13] employs a message-passing algorithm to integrate gene expression data with other data types for GRN inference, and KBoost [14] uses Kernel Principal Component Regression (KPCR) combined with boosting techniques, optionally incorporating known TF targets as prior information to infer GRNs.

We now delve into the specifics of how each of these methods performs under various settings, starting with GENIE3 and GRNBoost2. GENIE3 [15] utilizes random forest regression to capture non-linear gene relationships, while GRNBoost2 [7] applies a gradient boosting algorithm augmented with early stopping for regularization. For inputs, these methods use a TF list file, which remains consistent across all runs, and specific GEX matrices that vary with the analysis strategy — GEX_{dEN} and GEX_{h64} for DiNA, and GEX_{mix} for CsNA. To ensure consistency and robustness, we ran each method 20 times using different initialization seeds (--num_seed set to 20). This approach helps to ensure reliable results by accounting for the stochastic variability of these algorithms. In post-processing, we computed the confidence scores of the inferred edges by taking both the mean and median of the importance scores across all iterations.

Inferelator (version 3) is a regression-based method that utilizes modules including Bayesian Best Subset Regression (BBSR), Elastic Net (ElasticNet), and StARS-LASSO [11]. We obtained its code from its GitHub repository (https://github.com/flatironinstitute/inferelator). In its workflow pipeline, we set the number of bootstraps to five to enhance inference robustness and disabled cross-validation due to the absence of any gold standard or gene/sample metadata. All other parameters were maintained at their default settings. Inferelator requires a list of transcription factors and specific GEX matrices, along with ChIP-seq derived binary matrices, the selection of which depends on the analysis strategy (CsNA or DiNA) being implemented.

PANDA [13] employs a message-passing approach to integrate gene expression data, protein-protein interaction (PPI) data, and transcription factor binding motifs (TFBM) to infer GRNs. For our benchmarking, we used PANDA with RNA-seq and ChIP-seq data, modifying it to replace ChIP-seq information instead of TFBM information. While GEX matrices were used directly, ChIP-seq data was reformatted from a Gene x TF matrix to an edge list format, with each entry having three columns: TF, Gene, and a binary score. This reformatted ChIP-seq data was then substituted for the TFBM argument in PANDA. Depending on whether the DiNA or CsNA strategy was being employed, we ran PANDA with this setup, testing both the default learning rate of 0.1 and a reduced rate of 0.01. Additionally, other parameters such as modeProcess were left at their default values to maintain consistency across runs. The PANDA code was sourced from its GitHub repository (https://github.com/netZoo/netZooPy/tree/master/netZooPy/panda).

NetREX [12] (GitHub link) is an algorithm that uses the concept of transcription factor activity (TFA) to infer GRNs through bootstrapping and the Proximal Alternative Linearized Maximization algorithm. For inputs, NetREX requires RNA-seq data and ChIP-seq data, for which we used our pre-prepared GEX matrices (gene x sample) and Prior matrices (gene x TF), as detailed in the Input Data Configuration section. We ran this algorithm with all parameters set to their default settings

to ensure consistency. Notably, parameters such as -k and -t, which control the edge percentages from the prior network and the edge ratio in the output network respectively, were set to 'None,' allowing the algorithm to determine these dynamically based on the input data.

MERLIN+Prior [10] (GitHub link), developed within a C++ framework, utilizes a probabilistic graphical model to infer GRN. While MERLIN+Prior accommodates both modular and per-gene information, it also offers a simpler per-gene inference configuration known as *PGG+Prior*, which can be activated by setting the modularity parameter r to 0, with the default being r=4. Both tools require a tab-delimited expression file, a list of regulators, and an output directory. They also support multiple prior networks through a configuration file, which lists each network's name, file path, and a confidence level. Each prior network file, specified in the configuration file, should be formatted as an edge list, where each row represents one interaction edge: the first column for the regulator's name, the second for the target gene's name, and the third for the confidence in that edge, with higher values indicating greater trust in the interaction. To meet this specification, we converted our earlier prepared binary matrices ($Prior_{h64}$, $Prior_{dEN}$, $Prior_{mix}$) into an edge list format. The list of TFs and GEX matrices we prepared earlier remain unmodified. After that, we created three separate configuration files with one row for each binary network, specifying the network's name, the path to the prior network, and setting the confidence level to 5. This prior parameter was empirically determined to achieve the best AUPR from the range tested using simulated network in a cross validation framework in the original study (β^{motif}=5) [10]. Even though both algorithms can accept continuous scores, we decided to use binary scores in our prior networks because the Distance p-values do not conform to the principle that a higher score indicates higher confidence. Depending on the strategy employed, CsNA or DiNA, we ran the algorithms with the corresponding inputs—for CsNA strategy, this included GEX_{mix} , $Prior_{mix}$, in edge list format, a configuration file for $Prior_{mix}$, and a TF list. Throughout, we adhered to the default parameters: setting the sparsity parameter ρ to -5 for likelihood calculations and a hierarchical clustering threshold h to 0.6.

KBoost [14], implemented in an R framework, employs Kernel Principal Component Regression (KPCR), boosting, and Bayesian Model Averaging (BMA) to probabilistically estimate gene regulatory interactions. For inputs, it requires GEX data to be formatted as a matrix with samples as rows and genes as columns (N x G1), with each gene standardized to a mean of zero and a variance of one. To satisfy this requirement, we adjusted the GEX matrices by transposing and standardizing them. For the prior network, KBoost accepts a matrix of genes by TFs (denoted by G2 x K) containing the prior probabilities of each interaction. KBoost requires that the number of genes in the GEX matrix (G1) must match the number of genes in the prior network (G2). However, in our setup, G1 includes 22 TFs whereas the prior network is structured for non-TF genes interacting with TFs, resulting in a mismatch where G2 falls short of G1 by 22. To resolve this, we expanded those prepared prior matrices—which originally included only non-TF genes interacting with TFs—to include all genes by appending zeros for the missing TF-to-TF interactions, thereby aligning the dimensions of our gene set with the TFs. Furthermore, due to KBoost's logarithmic transformation of prior probabilities, we avoided computational errors by adjusting values of 1 to 0.9999999 and 0 to e^{-10} which we refer to as the "defaultPrior". Additionally, to introduce a deliberate uncertainty that helps in differentiating the posterior from

the "defaultPrior", we established another version of prior matrices: "fuzzyPrior", where values of 1 are set to 0.75 and values of 0 are set to 0.25. These adjustments, in accordance with the methodology outlined in the KBoost paper, account for the inherent probabilistic nature of gene regulatory interactions, thereby providing a more nuanced prior that encapsulates the potential uncertainties within the network.

iRafNet [8], implemented in an R framework, advances the GENIE3 methodology by introducing a unified integrative algorithm to the random forest model. This integrative approach allows iRafNet to accommodate diverse data types, including gene knock-down experiments and protein-protein interactions. For inputs, iRafNet requires expression data formatted with samples in rows and genes in columns, as well as a prior network. We adapted our pre-prepared GEX matrices by transposing them to align with the required sample-by-gene format and standardized each gene to have a mean of 0 and variance of 1. The iRafNet methodology provides various options for constructing the prior weights matrix, from symmetric matrices based on protein-protein interactions to directed weights derived from time-series and knockout data. To fit these specifications, we expanded our prior matrices (originally gene by TF) to a size of $(n_{gene} + n_{TF}) \times (n_{gene} + n_{TF})$, and filled the missing entries with zeros. This adjustment preserves the directed structure of gene-TF relationships and avoids unfounded assumptions of symmetry. For parameters, we maintained the default ntree setting of 1000 trees for the random forest, ensuring robust model performance. However, we customized the mtry parameter, which determines the number of potential regulators sampled at each tree node, setting it to 22 to specifically match the 22 transcription factors of interest in our study.

InPheRNo [9], a computational method built around a probabilistic graphical model, utilizes p-values of TF-gene and gene-phenotype associations, alongside a list of human transcription factors, to infer phenotype-relevant GRNs. Distinct from other methods we've evaluated, InPheRNo does not rely on DiNA or CsNA strategies to integrate phenotype information, as it incorporates this information explicitly in its PGM. We configured InPheRNo to run with the parameter --max_num_tf set to 22, corresponding to the number of TFs identified in our study. Additionally, we maintained the L1 ratio (of the elastic net algorithm) at the default setting of 0.5.

Establishing the ground truth GRNs associated with endoderm differentiation

To rigorously evaluate the performance of InPheRNo-ChIP, we established phenotype-specific ground truth networks focused on the role of three endoderm markers in the differentiation of hESCs toward dEN and endoderm's sub-lineages. These networks, focused on interactions of three TFs (*FOXA2*, *SMAD2*, and *SOX17*) with their target genes, were derived from a comprehensive experimental scRNA-seq CRISPRi study by Genga et al. [16], focused on the dynamic regulatory landscape during hESC to endoderm lineage transition. To construct the *FOXA2*-oriented network, we directly sourced regulatory targets of *FOXA2* from their Supplementary Table S4 [16], employing a Bonferroni correction threshold of <0.05, corresponding to genes that were differentially expressed as a result of *FOXA2* knockdown.

Unfortunately, the targets of *SMAD2* and *SOX17* were not readily available. To identify their target genes, we reanalyzed their data and replicated the clusters reported in the original article, allowing us to identify differentially expressed genes associated with the knockdown of each TF. As shown in their Figures 2A-2B, their cluster 2 was enriched with *SMAD2* gRNA cells and their cluster 3 was enriched with *SOX17* gRNA cells, an observation that we used to form *SMAD2*-oriented and *SOX17*-oriented networks, detailed below.

We utilized the R code available in the Maehr Lab GitHub repository (https://github.com/maehrlab/de_screen_analysis) and downloaded the scRNA-seq data from the GEO repository (accession GSE127202). Focusing on four specific samples (GSM3630200-203), which corresponded to the original study's clusters, we implemented their quality control measures (filtering out low-quality cells with fewer than 200 gene counts or a doublet-modelling log10 likelihood ratio above 0.2 and retaining genes present in at least two cells). We then performed principal component analysis. Using the first 15 principal components as input, we employed the 'FindClusters()' function with a resolution parameter of 0.075 to identify cell clusters using a shared nearest-neighbor (SNN) graph. Our results closely matched those reported in the original study, with Cluster 0 encapsulating differentiated endoderm cells, Cluster 1 including cells that halted early in the differentiation pathway, Cluster 2 comprising cells affected by post-*SMAD* signaling perturbations, and Cluster 3 containing cells transitioning into the mesoendoderm lineage.

For the *SMAD2*-oriented network, we utilized the 'FindMarkers()' function with the Wilcoxon Rank Sum test to identify differentially expressed genes between Clusters 2 and 0. After Bonferroni correction (bonf <0.05, |logFC| >0.25), these DE genes were used to construct the network. Similarly, recognizing the expression patterns of *SOX17* in Clusters 0 and 3, we applied the same method to identify differentially expressed genes between those clusters for the *SOX17*-oriented network.

Performance Evaluation Metrics for GRN Reconstructions

In this study, we assessed the predictive precision of GRN inference methodologies with a suite of computational metrics such as the Area Under the Precision-Recall Curve (AUPRC) — including its normalized comparison to a random predictor — and Early Precision at K (denoted as EarlyPrec@K in supplementary Table S4) along with F-beta metrics. A crucial step in our analysis was defining a 'shared universe' of genes, which is the overlap between the ground truth network and the genes from our RNA-seq and ChIP-seq data. This common gene set ensured that our evaluation was based on genes represented both in the experimental data and the prediction models.

AUPRC, AUPR and their variants: In the evaluation of our benchmarking algorithms, we prioritized the Area Under the Precision-Recall Curve (AUPR) over the Area Under the Receiver Operating Characteristic (AUROC). This approach is particularly suited for GRNs, characterized by their sparse nature, where the accurate detection of rare true positive interactions is more critical than merely distinguishing true positives from negatives. The AUPR's sensitivity in sparse data scenarios makes it a more reliable metric for assessing the true efficacy of an algorithm in

discerning genuine regulatory relationships. To compute the AUPRC, we first generated a series of precision and recall values at various threshold levels, utilizing the `precision_recall_curve` function from the sklearn.metrics library. These values were then integrated using the auc function to obtain the AUPRC. Our method extends beyond merely calculating the AUPRC; we also compared it against the expected performance of a random predictor. The random predictor's AUPRC is determined by the proportion of positive instances in the ground truth network.

EarlyPrec@K: We adopted this metric to focus on the precision of the top 'K' interactions predicted by the GRN inference methods, ranked according to the importance scores assigned by the algorithms ranking from the largest positive score to zero or negative scores. By examining various 'K' values, from 10 to 100 in increments of 10, we evaluated the robustness of the methods' predictions at different list depths.

F-beta scores: We utilized F0.5, F1, and F2 scores, to evaluate the balance between precision and recall in our methods.

- F0.5 emphasizes precision over recall, useful in limiting false positives.
- F1 equally balances precision and recall, providing a balanced evaluation.
- F2 focuses more on recall, useful when identifying true positives is critical.

Gene set enrichment analysis using Enrichr platform

We used Enrichr platform [17] to perform gene set enrichment analysis. As inputs, we selected top 12 TFs in the Table 1 and pasted their target genes identified by InPheRNo-ChIP into the platform. These gene sets were mapped to cell types using the Human Gene Atlas, with the results provided in Supplementary Table S5. Enrichr offers several ranking methods for terms, including Fisher's exact test (FET) p-value, adjusted p-value, odds ratio, and combined score. The combined score, which multiplies the log-transformed p-value by a rank deviation z-score (Combined Score = log(p-value) * z-score), has been reported to produce the most accurate rankings [17]. For downstream analysis, we applied the combined score for filtering, selecting terms that exceeded the 85th percentile threshold for each TF.

Supplementary Tables

Supplementary Table S1: Detailed sample information. This information includes their source and relevant experimental details. Sheets 2 through 4 provide detailed sample information for the RNA-seq datasets utilized in this study, extracted from GEO Series_matrix files. These sheets include common columns such as Sample_title, Sample_source_name, Sample_organism, and Sample_molecule. Additionally, they detail study-specific information like experimental conditions towards the end of each tab. Sheet 6 includes 28 untreated (control) samples that we used in this study. Sheet 5 includes ChIP-seq data from the GEO repository, covering a wide range of cell types, with our analysis specifically focused on the dEN and hESC lineages to align with our study's objectives. The detailed tables can be accessed in the file "TableS1.xlsx".

Supplementary Table S2: Details of RNA-seq analysis. The file provides sheets with information pertaining to the first two steps of InPheRNo-ChIP pipeline. "Voom-zScored" Tab corresponds to Step 2 of the InPheRNo-ChIP methodology, containing z-normalized gene expression data prepared using Limma's voom function, followed by an inverse-quantile normalization. "DE_all" and "DE_logFC2_FDR0.01" Tabs relate to Step 1, showcasing results from the differential expression analysis conducted using EdgeR. They detail significant gene expression changes between hESC and dEN conditions, processed to extract gene-phenotype associations with stringent filters applied (BH-FDR < 0.01 and $|logFC| > 2$). The detailed tables can be accessed in the file "TableS2.xlsx".

Supplementary Table S3: The reconstructed lineage-relevant GRN using InPheRNo-ChIP. The RnP tab, an ablated variant, uses TF-gene and gene-phenotype associations from RNA-seq data to infer the GRN. The CnP tab, another ablated variant, integrates TF-gene associations from ChIP-seq with gene-phenotype associations from RNA-seq to infer the GRN. The RnCnP tab represents the complete model, inferring a network by incorporating TF-gene associations from both RNA-seq and ChIP-seq, alongside gene-phenotype associations from RNA-seq. The network visualized in Figure 2 of the manuscript is based on the RnCnP tab. The detailed tables can be accessed in the file "TableS3.xlsx".

Supplementary Table S4: Detailed performance of GRN inference methods. This file contains the result of benchmarking of various GRN inference algorithms utilized in the InPheRNo-ChIP study, spanning three distinct tabs. The "TF-level" tab evaluates algorithms based on the TF-oriented ground truth networks, which were derived from a perturb-seq study. This tab supports Figure 4. The "edge-level" tab examines the performance of these algorithms across the entire network, assessing their effectiveness in modeling interactions at a broader scale, as detailed in Figure 5. The "configs" tab provides detailed configurations for each algorithm, listing elements like algorithm names, input data modalities, core ideas, and specific parameters. The detailed tables can be accessed in the file "TableS4.xlsx".

Supplementary Table S5: Enrichr gene set enrichment analysis results. This file ("TableS5.xlsx") contains the results of gene set enrichment analysis performed using the Enrichr platform on the top 12 TFs identified by InPheRNo-ChIP and their corresponding high-confidence target genes (confidence score > 0.5). The file includes 12 tabs, each corresponding to one of the TFs, with detailed information on enriched terms, including overlap, P-value, adjusted P-value, odds ratio, combined score, and the list of genes contributing to the enrichment. The results highlight the associations between the TFs and various cell types, focusing on terms passing the 85th percentile threshold of combined scores, as explained in the Methods section. These data were used to generate Figure 5 in the main manuscript.

Supplementary Figures

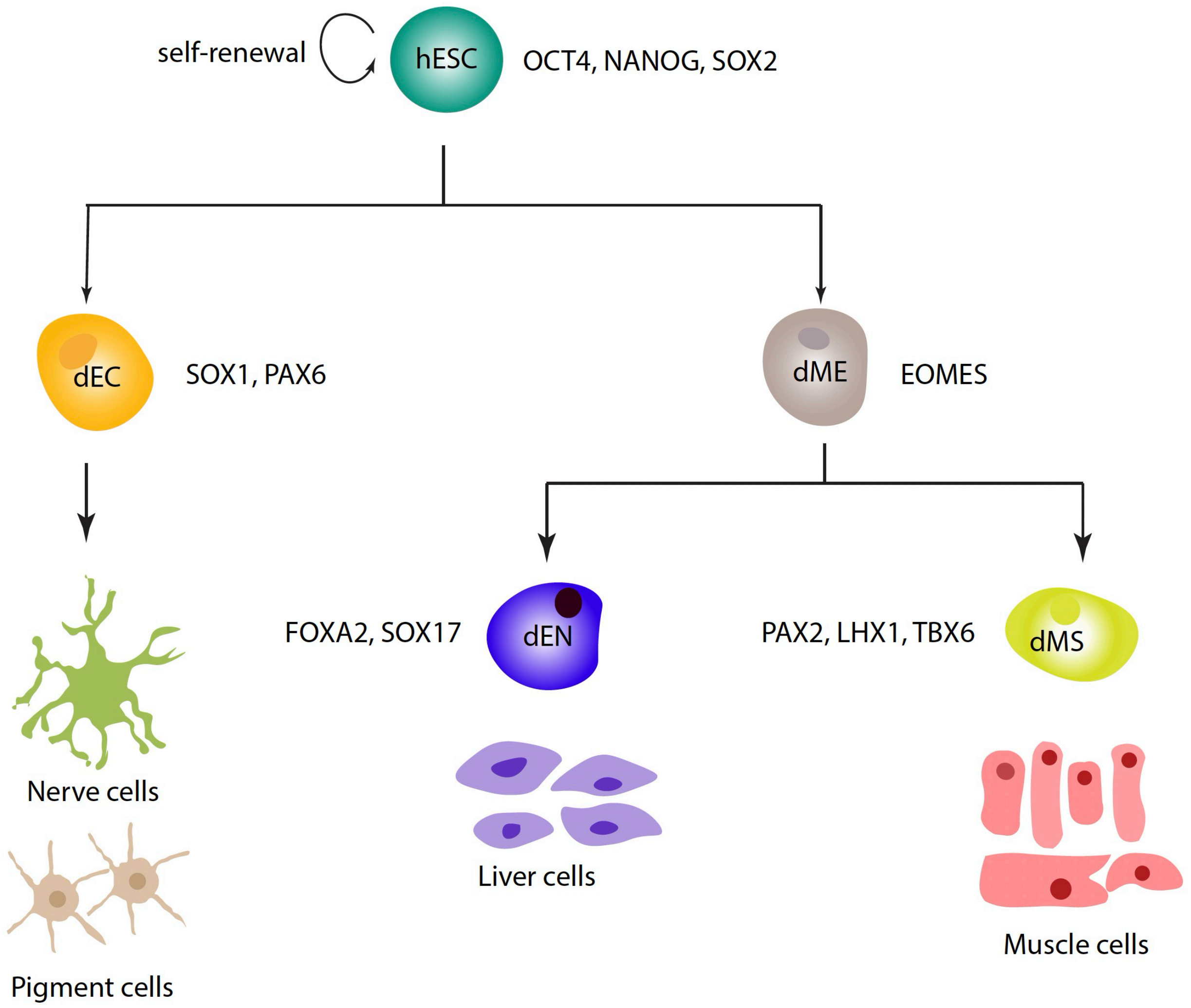

Supplementary Figure S1. Schematic of human embryonic stem cell (hESC) differentiation and lineage specification. This diagram depicts the differentiation of hESCs into the three primary germ layers: ectoderm (EC), mesoderm (dMS), and definitive endoderm (dEN). Key marker genes associated with each developmental stage are highlighted. Pluripotent hESCs express OCT4, NANOG, and SOX2, enabling self-renewal. Differentiation is marked by the emergence of lineage-specific genes: SOX1 and PAX6 for ectoderm, EOMES for mesoderm, and FOXA2 and SOX17 for definitive endoderm. The endoderm lineage gives rise to organs such as the liver, lungs, pancreas, and gut. PAX2, LHX1, and TBX6 indicate mesoderm differentiation, leading to diverse cell types including muscle, kidney, and bone. This illustration provides a simplified overview of the complex gene regulation governing cell fate decisions during early human development and The focus of the study is on the transition from hESC to definitive endoderm (dEN).

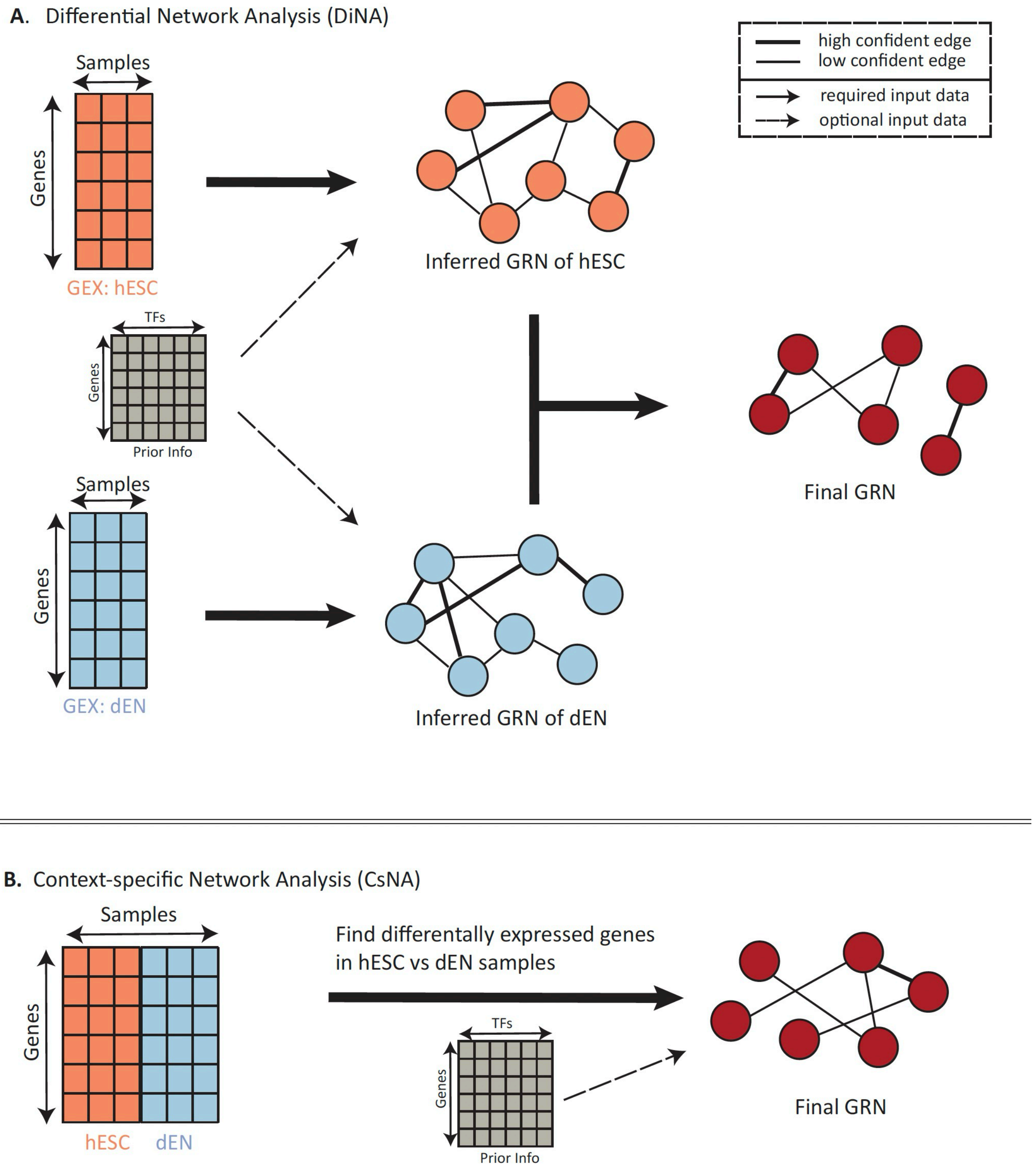

Supplementary Figure S2. Schematic Overview of Network Analysis Methods Employed in GRN Inference: Differential Network Analysis (DiNA) in Panel A and Context-Specific Network Analysis (CsNA) in Panel B.

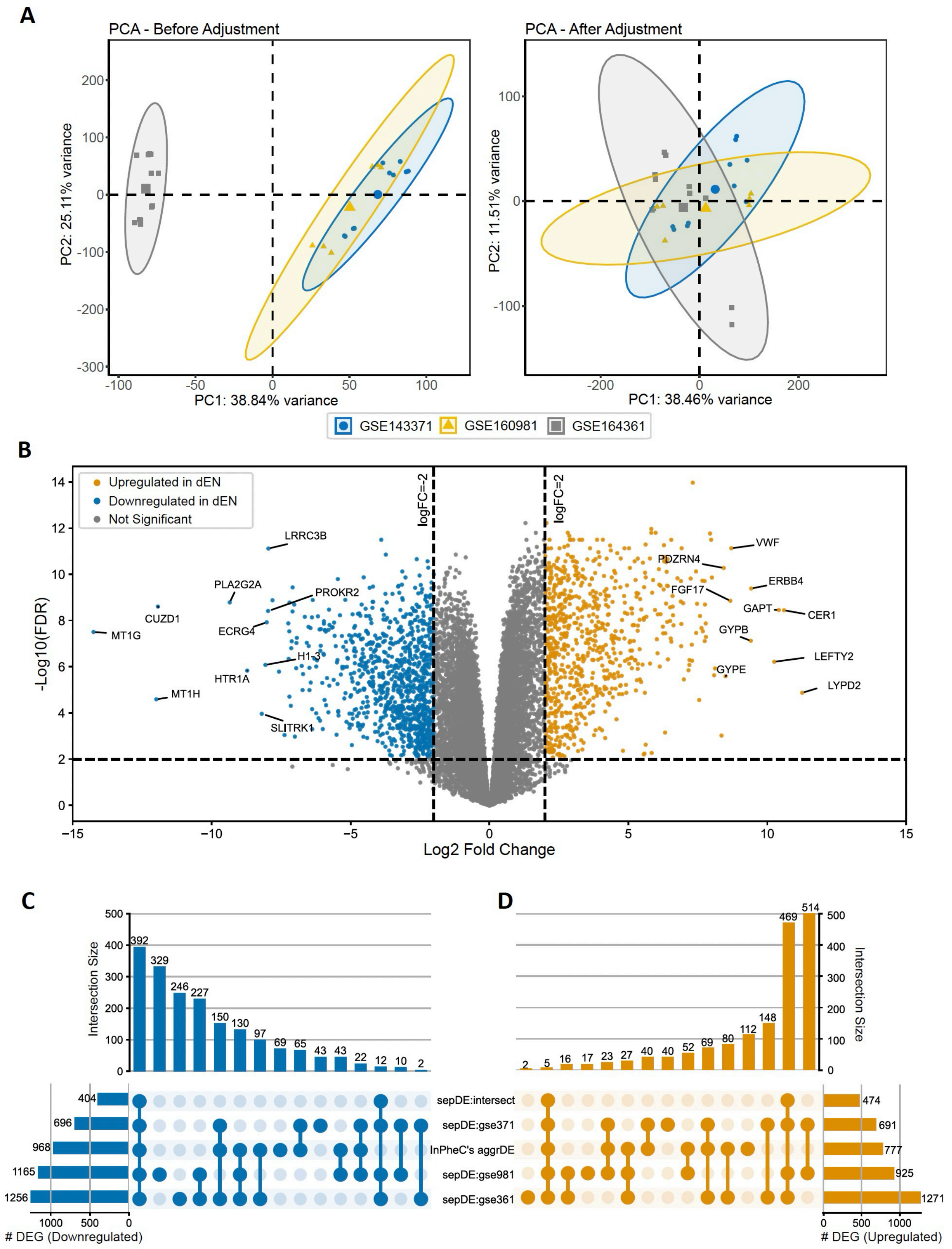

Supplementary Figure S3. Preprocessing, integration and differential expression analysis using RNA-seq datasets of hESC-to-dEN differentiation. Panel A corresponds to principal component

analysis (PCA) plots illustrating the removal of batch effects in the RNA-seq data of hESC and dEN samples using ComBat-seq. Ellipses represent confidence intervals around the group centroids, with colors corresponding to different datasets as labeled in the legend. Panel B shows the volcano plot for differential expression analysis of the aggregated datasets post batch effect removal. Points are colored to denote genes that are upregulated in dEN relative to hESC (gold), downregulated (blue), or showing no significant change (gray). Notable genes with significant fold changes are labeled. Panel C and D show UpSet plots for upregulated and downregulated genes. Each of them shows the overlap (intersection) of differentially expressed genes found in various datasets. Labels of the form "sepDE:GSExxx" refer to separate DE analyses conducted on individual datasets. "sepDE:intersect" represents the intersection of up- or down-regulated genes identified across all three separate DE analyses. "inpherC's aggrDE" signifies aggregated DE results from the volcano plot in panel B, which was used in this study.

Reference

1. Hu, H., et al., *AnimalTFDB 3.0: a comprehensive resource for annotation and prediction of animal transcription factors.* Nucleic acids research, 2019. **47**(D1): p. D33-D38.
2. Amemiya, H.M., A. Kundaje, and A.P. Boyle, *The ENCODE blacklist: identification of problematic regions of the genome.* Scientific reports, 2019. **9**(1): p. 9354.
3. Robinson, M.D., D.J. McCarthy, and G.K. Smyth, *edgeR: a Bioconductor package for differential expression analysis of digital gene expression data.* bioinformatics, 2010. **26**(1): p. 139-140.
4. Zhang, Y., G. Parmigiani, and W.E. Johnson, *ComBat-seq: batch effect adjustment for RNA-seq count data.* NAR genomics and bioinformatics, 2020. **2**(3): p. lqaa078.
5. Landt, S.G., et al., *ChIP-seq guidelines and practices of the ENCODE and modENCODE consortia.* Genome research, 2012. **22**(9): p. 1813-1831.
6. Quinlan, A.R. and I.M. Hall, *BEDTools: a flexible suite of utilities for comparing genomic features.* Bioinformatics, 2010. **26**(6): p. 841-842.
7. Moerman, T., et al., *GRNBoost2 and Arboreto: efficient and scalable inference of gene regulatory networks.* Bioinformatics, 2019. **35**(12): p. 2159-2161.
8. Petralia, F., et al., *Integrative random forest for gene regulatory network inference.* Bioinformatics, 2015. **31**(12): p. i197-i205.
9. Emad, A. and S. Sinha, *Inference of phenotype-relevant transcriptional regulatory networks elucidates cancer type-specific regulatory mechanisms in a pan-cancer study.* NPJ systems biology and applications, 2021. **7**(1): p. 9.
10. Siahpirani, A.F. and S. Roy, *A prior-based integrative framework for functional transcriptional regulatory network inference.* Nucleic acids research, 2017. **45**(4): p. e21-e21.
11. Skok Gibbs, C., et al., *High-performance single-cell gene regulatory network inference at scale: the Inferelator 3.0.* Bioinformatics, 2022. **38**(9): p. 2519-2528.
12. Wang, Y., et al., *Reprogramming of regulatory network using expression uncovers sex-specific gene regulation in Drosophila.* Nature Communications, 2018. **9**(1): p. 4061.
13. Glass, K., et al., *Passing messages between biological networks to refine predicted interactions.* PloS one, 2013. **8**(5): p. e64832.
14. Iglesias-Martinez, L.F., B. De Kegel, and W. Kolch, *KBoost: a new method to infer gene regulatory networks from gene expression data.* Scientific Reports, 2021. **11**(1): p. 15461.
15. Huynh-Thu, V.A., et al., *Inferring regulatory networks from expression data using tree-based methods.* PloS one, 2010. **5**(9): p. e12776.
16. Genga, R.M., et al., *Single-cell RNA-sequencing-based CRISPRi screening resolves molecular drivers of early human endoderm development.* Cell reports, 2019. **27**(3): p. 708-718. e10.
17. Kuleshov, M.V., et al., *Enrichr: a comprehensive gene set enrichment analysis web server 2016 update.* Nucleic acids research, 2016. **44**(W1): p. W90-W97.
